# Supplementary material for: The impact of sex on gene expression in the brain of schizophrenic patients: a systematic review and meta-analysis of transcriptomic studies
Source: Biol Sex Differ. 2024 Jul 27;15:59. doi: 10.1186/s13293-024-00635-x (PMC11282642; doi:10.1186/s13293-024-00635-x)
Supplement: Supplementary file 15 — Supplementary Material 15 [file 13293_2024_635_MOESM15_ESM.docx]

**Sup. table 1:** Summary of studies selected

**Sup. table 2:** Characteristics of samples used in meta-analyses

**Sup. table 3:** Differentially expressed genes in the meta-analysis of PFC studies

**Sup. table 4:** Pairs of interacting proteins significantly altered in males in the meta-analysis of PFC studies

**Sup. table 5:** Altered biological process GO terms in the meta-analysis of PFC studies

**Sup. table 6:** Regulons analyzed in the meta-analysis of PFC studies

**Sup. table 7:** Differentially expressed genes in the meta-analysis of hippocampus studies

**Sup. table 8:** Pairs of interacting proteins significantly altered in males in the meta-analysis of hippocampus studies

**Sup. table 9:** Pairs of interacting proteins significantly altered in females in the meta-analysis of hippocampus studies

**Sup. table 10:** Altered biological process GO terms in the meta-analysis of hippocampus studies

**Sup. table 11:** Regulons analyzed in the meta-analysis of hippocampus studies

**Sup. table 12:** Differentially expressed genes in the meta-analysis of all studies

**Sup. table 13:** Altered biological process GO terms in the meta-analysis of all studies

**Sup. table 14:** Common biological process GO terms altered in female hippocampus and male PFC of schizophrenic patients
